# Insight into mechanism of oxidative DNA damage in angiomyolipomas from TSC patients

**DOI:** 10.1186/1476-4598-8-13

**Published:** 2009-03-05

**Authors:** Samy L Habib

**Affiliations:** 1 Geriatric Research Education and Clinical Center, South Texas Veterans Healthcare System, San Antonio, TX, USA; 2 Department of Medicine, University of Texas Health Science Center, San Antonio, Texas 78229, USA

## Abstract

**Background:**

The tuberous sclerosis complex (TSC) is caused by defects in one of two tumor suppressor genes, TSC-1 or TSC-2. TSC-2 gene encodes tuberin, a protein involved in the pathogenesis of kidney tumors, both angiomyolipomas and renal cell carcinomas. Loss of heterozygosity at the 8-oxoG-DNA glycosylase (OGG1) allele is found in human kidney clear cell carcinoma identifying loss of OGG1 function as a possible contributor to tumorigenesis in the kidney. Tuberin regulates OGG1 through the transcription factor NF-YA in cultured cells. The purpose of this study is to determine the effect of tuberin-deficiency on OGG1 protein and mRNA levels as well as on 8-oxodG levels in kidney tumors from patients with TSC. In addition we evaluated the phophorylation level of downstream targets of mTOR, phospho-S70K, in kidney tumor tissue from TSC patients.

**Results:**

Kidney angiomyolipoma tissue from TSC patients expresses significant levels of phopho-tuberin and low levels of tuberin compared to control kidney tissue. The increase in tuberin phosphorylation and the decrease tuberin expression are associated with decrease in OGG1 protein and mRNA levels in tumor samples compared to normal kidney samples. The decrease OGG1 expression is also associated with significant decrease in the transcription factor, NF-YA, expression in tumor samples compared to normal tissues. In addition, the levels of 8-oxodG are 4-fold higher in tumors compared to control samples. The significant increase of phospho-tuberin expression is associated with increase phosphorylation of S6K in tumor samples compared to controls. Cyclin D1 expression is also 3-fold higher in increase in the tumor tissues compared to normal kidney tissues.

**Conclusion:**

These data indicate that tuberin deficiency in angiomyolipoma enhances mTOR activation by phosphorylation of S6K and downregulation of protein and mRNA expression of OGG1 resulted in accumulation of oxidized DNA in patients with TSC. These data suggest that tuberin and OGG1 are important proteins in the pathogenesis of angiomyolipoma in TSC patients.

## Background

Tuberous sclerosis complex (TSC) is an autosomal dominant genetic disorder associated with tumors in many organs, particularly angiomyolipoma in the kidneys and renal cell carcinoma (RCC). TSC affects about 1 million individuals worldwide, with an estimated prevalence of up to 1 in 6,000 newborns [1]. Loss of heterozygosity (LOH) at the *TSC1 *or *TSC2 *loci has been detected in *TSC*-associated hamartomas and renal cell carcinoma (RCC) as well as in sporadic tumors of non-TSC patients [2]. Multicentric angiomyolipomas are much more common in patients with TSC than RCCs, but may nonetheless have similar underlying genetic basis at early steps in their genesis and/or progression, specifically in the setting of tuberin deficiency. Renal angiomyolipomas (ALMs) associated with TSC tend to be larger, bilateral, multifocal and present at a younger age compared with sporadic forms [2]. ALMs are generally benign tumors, which are composed of smooth muscle, fat, and blood vessels [2]. Kidney cancer development is rare in TSC, occurring in only 2–3% of all patients [3-5]. The *TSC2 *gene product (tuberin) is a tumor suppressor protein whose absence or inactivation is associated with several defects such as abnormal cellular migration, proliferation, and differentiation [6,7]. Tuberin expression was initially induced following acute renal injury, suggesting that the *TSC2 *gene may function as an acute-phase response gene, limiting the proliferative response after injury [8]. Tuberin is a target of both serine/threonine and tyrosine kinases [9,10]. Most recently, tuberin has been shown to be a target for phosphorylation by several kinases including Akt [11]. Akt directly phosphorylates and inactivates TSC2 on Ser 924, Thr 1462 and Thr 1518. These phosphorylations by Akt disrupt the TSC1-TSC2 complex and disturb the subcellular localization of TSC1 and TSC2 [11].

Oxidative DNA damage has been implicated in carcinogenesis, ageing and several age-related degenerative diseases [12,13]. 8-Oxo-deoxyguanine (8-oxo-dG) is a quantitatively major form of oxidative DNA damage [11,12], inducing mainly G to T and A to C substitutions [13]. 8-Oxo-dG in DNA is repaired primarily via the DNA base excision repair pathway. The gene coding for the DNA repair enzyme that recognizes and excises 8-oxo-dG is 8-oxoG-DNA glycosylase (OGG1) [14]. Deficiency in DNA repair enzyme OGG1 has important functional consequences, compromising the ability of cells to repair DNA [14,15]. OGG1 is a functional, but not structural, analogue of the bacterial Fpg protein. OGG1 deficiency in yeast, as well as Fpg deficiency in bacteria, results in a spontaneous mutator phenotype [16]. The steady-state levels of 8-oxoG, which reflect the balance between its continuous generation and removal, are significantly higher in livers of *OGG1*^-/- ^mice compared to wild-type animals [17]. Increased susceptibility to mutagens and impaired DNA repair can contribute to the genomic instability and in consequence to cancer. The *OGG1 *gene is somatically mutated in some cancer cells and is highly polymorphic among humans [18]. Loss of heterozygosity at the *OGG1 *allele, located on chromosome 3p25, is found in 85% of 99 human kidney clear cell carcinoma samples, identifying loss of OGG1 function as a possible consequence of multistep carcinogenesis in the kidney [19]. A nuclear factor-YA (NF-YA) has been identified as a transcription factor that binds to a consensus sequence in the OGG1 promoter [20]. NF-Y is a ubiquitous transcription factor that specifically recognizes a CCAAT box motif and regulates *hOGG1 *expression as well as genes that regulate development and cell cycle [20].

The constitutive expression of OGG1 in heterozygous Eker rat (TSC2^+/-^) kidneys is lower than in wild-type rats [21,22] suggesting that these proteins may be functionally linked. Downregulation of tuberin in human renal epithelial cells causes in a marked decrease in the abundance of OGG1 [23]. Mouse embryonic fibroblasts deficient in tuberin (*TSC2*^-/- ^and *TSC2*^+/-^) also express very low levels of OGG1 mRNA and protein and undetectable OGG1 activity accompanied by accumulation of 8-oxodG [23]. The present study was conducted to investigate the effect of tuberin deficiency on phosphorylation of S6Kinase (downstream targets of mTOR) and on OGG1 expression and 8-oxodG levels in kidney tumors from TSC patients.

## Results

### Tuberin phosphorylation is increased and is associated with decreased tuberin and OGG1 expression in kidney angiomyolipoma tissue

**Figure 1:**
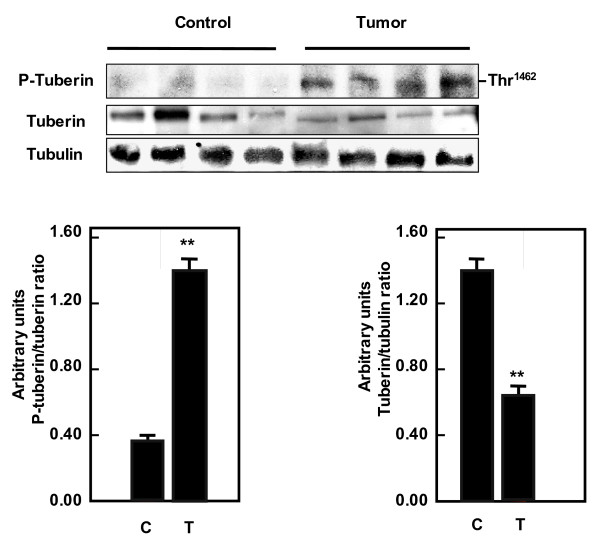
**Increased phosphorylation of tuberin and decreased total tuberin in kidney tumors**. Immunoblot analysis of phospho-tuberin (Thr 1462) and tuberin protein expression in normal kidney samples and tumor kidney from patients with tuberous sclerosis. Increased tuberin phosphorylation is associated with decreased total tuberin expression in tumor tissue compared to normal tissue. Tubulin was used as loading control. Histograms represent means ± SE normal kidney samples and kidney tumors from patients with tuberous sclerosis. Significant difference between control and tumor groups is indicated by **P < 0.01.

**Figure 2:**
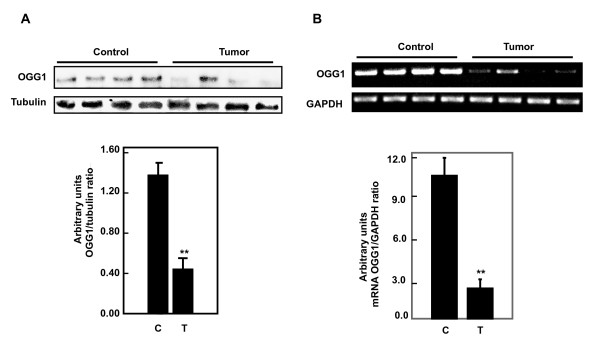
**Tuberin deficiency is associated with decreased protein and mRNA of OGG1 expression in kidney tumors**. (A) Immunoblot analysis of OGG1 protein expression in normal kidney and kidney tumor from patients with tuberous sclerosis. Decreased OGG1 is associated with decreased tuberin expression in tumor tissue compared to normal tissue. Tubulin was used as loading control. (B) RNA was extracted from kidney or tumor tissue and its integrity tested by formaldehyde/agarose gel electrophoresis. RTPCR was performed using one μg RNA from each sample to synthesize first and second strand DNA. PCR products were analyzed on ethidium bromide stained gel. Band intensity was quantified for each sample on an image analyzer and the ratio of OGG1 to actin was calculated. Histograms represent means ± SE normal kidney samples and kidney tumors from patients with tuberous sclerosis. Significant difference between control and tumor groups is indicated by **P < 0.01.

To determine the relevance of our findings in cell culture [23] and rodents [21,22] to humans, phopho-tuberin, total tuberin and OGG1 expression was assessed in tissue homogenates of control kidney tissue and kidney tumors from patients with tuberous sclerosis. There is increase in Thr 1462 phospho-tuberin in the tumor tissue compared to control kidney tissue (Figure 1). The increase tuberin phophorylation is associated with a decrease in tuberin levels (Figure 1). In addition decrease tuberin expression is associated with significant decrease in the mRNA and protein levels of OGG1 in tumor tissue compared to control tissue (Figure 2A and Figure 2B).

### Downregulation of OGG1 mRNA is associated with decreased NF-YA expression in kidney angiomyolipoma tissue

**Figure 3:**
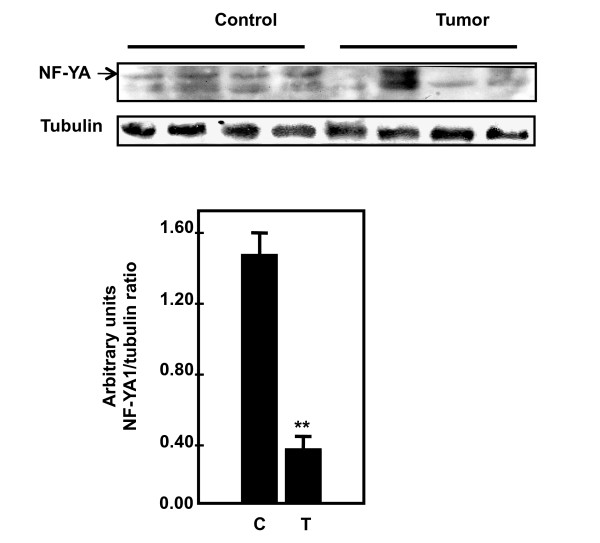
**Downregulation of OGG1 mRNA is associated with decreased NF-YA protein expression in kidney tumors**. Immunoblot analysis of NF-YA expression in normal kidney and kidney tumor from patients with tuberous sclerosis. Decreased OGG1 is associated with decreased NF-YA expression in tumor tissue compared to normal tissue. Tubulin was used as loading control. Histograms represent means ± SE normal kidney samples and tumors kidney from patients with tuberous sclerosis. Significant difference between control and tumor groups is indicated by **P < 0.01.

The decrease in *OGG1 *mRNA in kidney tumor samples suggests that decreased transcription is one potential mechanism responsible for downregulation of OGG1 protein. NF-YA is a transcription factor that regulates expression of OGG1. Our recent published data show that deficiency of tuberin in null and heterozygous mouse embryonic fibroblast cells is associated with decreased in NF-YA expression [23]. Protein expression of NF-YA in normal and tumor kidney samples was analyzed by western blot. NFYA expression is significantly decreased in most tumor kidney samples compared to control kidney samples (Figure 3).

### Accumulation of 8-OxodG in kidney angiomyolipoma tissue

**Figure 4:**
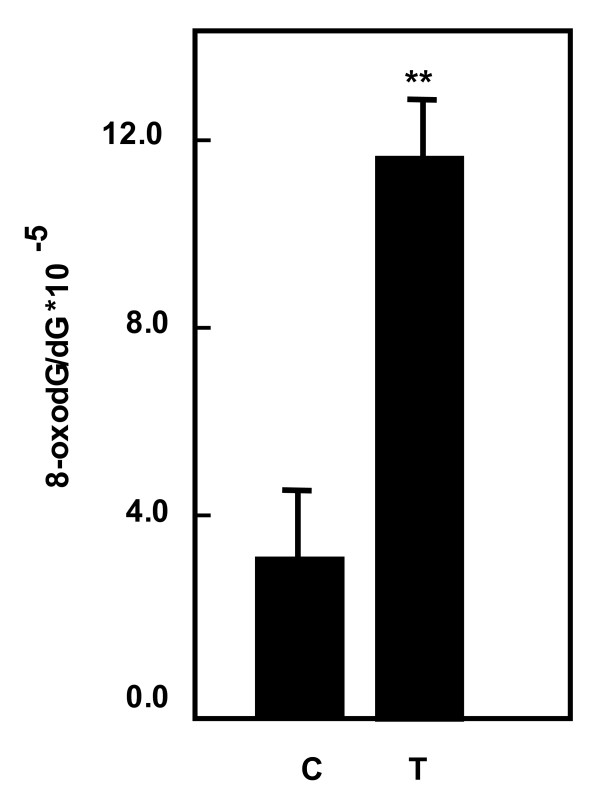
**Increased in 8-oxodG levels in kidney tumors**. DNA was extracted from normal kidney and kidney tumor from patients with tuberous sclerosis. Detection of dG and 8-oxodG was performed by HPLC-EC analysis. Authentic standards of 8-oxodG and dG were analyzed simultaneously. Standard curves for dG and 8-oxodG were prepared and quantitated by linear regression analyses Histograms represent means ± SE normal kidney samples and kidney tumors from patients with tuberous sclerosis. Significant difference between control and tumor groups is indicated by **P < 0.01.

Tuberin deficiency in *Tsc2*-null mouse embryonic fibroblast cells and in kidney Eker rat results in the accumulation of significant amounts of 8-oxodG [22,23]. To determine if the decrease in OGG1 is associated with 8-oxodG accumulation in angiomyolipomas, we quantified 8-oxodG by HPLC in control and tumor kidney samples. The amount of 8-oxodG in DNA extracted from tumor tissue is 4-fold higher in tumors than in control tissue (Figure 4).

### Activation of mTOR in kidney angiomyolipoma tissue

**Figure 5:**
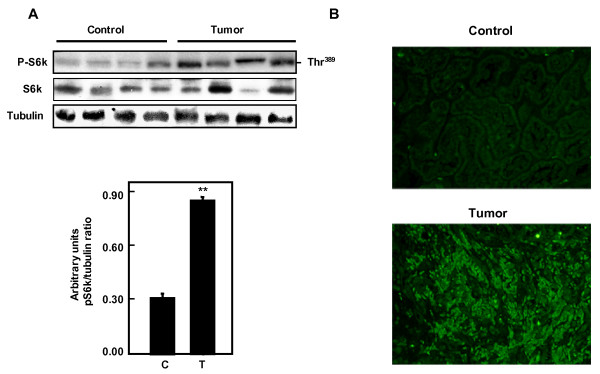
**Increased phosphoryaltion of S6K in kidney tumors**. (A) Immunoblot and (B) immunostaining analysis of phopho-S6K (Thr-389) and total S6K in normal kidney samples and kidney tumor from patients with tuberous sclerosis. Phosphorylation of S6K was significantly higher in tumor kidney tissue compared to normal kidney confirming the increase of mTOR activity. Tubulin was used as loading control. Histograms represent means ± SE normal kidney samples and kidney tumors from patients with tuberous sclerosis. Significant difference between control and tumor groups is indicated by **P < 0.01.

To determine if tuberin phosphorylation activates mTOR in tumor kidney tissue, phospho S6k expression was measured in kidney tissue homogenates of normal and tumor tissue from patients with TSC by western blot. Phosphorylation of S6K on Thr^389 ^is significantly increased in tumor tissues compared to normal samples (Figure 5A). Immunostaining of phosp-S6K is significantly increased in tumor kidney tissue compared to normal kidney confirming the increase in mTOR activity (Figure 5B).

### Tuberin deficiency is associated with increased cyclin D1 expression in kidney angiomyolipoma tissue

**Figure 6:**
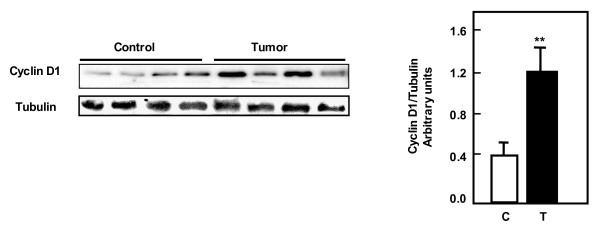
**Tuberin deficiency is associated with increased cyclin D1 expression in kidney tumors**. Immunoblot analysis of cyclin D1 in normal kidney samples and tumor from patients with tuberous sclerosis. Cyclin D1 was significantly higher in tumor kidney tissue compared to normal kidney. Tubulin was used as loading control. Histograms represent means ± SE normal kidney samples and kidney tumors from patients with tuberous sclerosis. Significant difference between control and tumor groups is indicated by **P < 0.01.

To determine the association between tuberin and cyclin D1, kidney tissue of control and tumor samples were analyzed by western blot. Cyclin D1 expression is significantly increased up to 3-fold in tumor tissues compared to control tissue indicating that tuberin is upstream of cyclin D1 (Figure 6).

## Discussion

This study is the first report to demonstrate that deficiency of tuberin in patients with tuberous sclerosis is associated with significant decrease of OGG1 and accumulation of 8-oxodG in angiomyolipomas suggesting that both proteins may play a major role in development of this kidney tumor. Deficiency and/or enhanced phosphorylation of tuberin on threonine 1462 results in its inactivation [19,10]. Our data demonstrate that tuberin phosphorylation on Thr^1462 ^residue in angiomyolipomas tissue is associated with deficiency of total protein. Phosphorylation of tuberin at this site affects its function through at least two mechanisms: first, phosphorylation decreases the activity of tuberin; second, phosphorylation destabilizes tuberin by disrupting the complex formation between hamartin and tuberin resulting in ubiquitination of free tuberin and its degradation by the proteosome [11]. Phosphorylation of tuberin by Akt also reduces the stability of tuberin and thereby releases its inhibitory function on p70S6K signaling [11].

The generation of oxidative DNA damage is counteracted in all species by specific repair mechanisms [15]. OGG1 is one of the major enzymes involved in the repair of 8-oxodG adducts in DNA and is highly expressed in kidney tissue. Loss of heterozygosity at the *OGG1 *allele was found in human kidney clear cell carcinoma, identifying loss of OGG1 function as a possible consequence of multistep carcinogenesis in the kidney [19]. We have previously shown that the constitutive expression of OGG1 in TSC2 heterozygous Eker rat (TSC2^+/-^) kidneys is lower than in wild-type rats [21]. We now find that decrease in tuberin protein expression in angiomyolipomas tissues is associated with a decrease in protein and mRNA expression of OGG1. Therefore, tuberin deficiency, through its phosphorylation is upstream of OGG1. The decrease in OGG1 expression in *TSC-2*^+/- ^rats has important functional consequences, compromising the ability of these animals to respond to oxidative stress [21]. The decrease in *OGG1 *mRNA in angiomyolipoma tissue suggests that decreased transcription is one potential mechanism responsible for downregulation of OGG1 protein. We have recently shown that suppression of renal OGG1 in tuberin deficient cells is mediated at least in part through the transcription factor, NF-YA, the major transcription factor that regulates the OGG1 gene expression [23]. In this study, NF-YA expression is decreased in angiomyolipoma tissue compared to control tissue suggesting that the decrease in OGG1 protein is due to decreased transcription.

The base excision pathway initiated by OGG1 represents the main defense against the mutagenic effects of 8-oxodG. Dysregulation of human DNA repair gene OGG1 is associated with an increased cancer risk. 8-OxodG induces mutation via misincorporation of DNA bases present in the unrepaired DNA adducts, or by slippage of DNA polymerase during replicative bypass. In this study, we demonstrate that 8-OxodG accumulates in angiomyolipomas tissue compared to normal tissue suggesting the deficiency of DNA repair OGG1. However our new published data show that loss of OGG1 expression in kidney tumor tissue from Eker rat resulted in the accumulation of significant amounts of 8-oxodG (unrepaired DNA lesions), suggesting that loss of tuberin is biologically relevant in affecting OGG1 [22]. We recently showed also that mouse embryonic fibroblasts deficient in tuberin (*Tsc2*^-/- ^and *Tsc2*^+/-^) had markedly decreased OGG1 mRNA and protein expression, as well as undetectable OGG1 activity accompanied by accumulation of 8-oxodG. [23]. In addition downregulation of tuberin in human renal epithelial cells using siRNA resulted in a marked decrease in the abundance of OGG1 [23]. Mice lacking a functional OGG1 protein accumulate abnormal levels of 8-oxodG in their genome and display a moderately elevated spontaneous mutation rate in nonproliferative tissues. Therefore, the antimutator function of OGG1 protein portrays *OGG1 *as a strong susceptibility candidate gene for tumors including angiomyolipoma.

The mammalian target of rapamycin (mTOR) serves a critical role in regulating the translational machinery that affects growth, proliferation, and differentiation, all of which are abnormally manifested in TSC lesions. The importance of mTOR pathway in human pathology is reflected in the overexpression of p70S6K in a subset of cancer and its correlation with a poor prognosis [24]. Activation of S6 Kinase and its target S6 ribosomal protein (S6) was demonstrated in cells lacking *TSC2 *expression [24]. Recent studies have shown that activation of mTOR in lymphangioleiomyomatosis-associated angiomyolipomas through phosphorylation of S6 protein at Ser235/236 [25]. Our data show that phosphorylation of S6 Kinase at Thr^389 ^was significantly higher in angiomyolipomas tissues compared to control samples indicating the increase of mTOR activity in tumor tissues.

Renal cortical tumors from the Eker rat heterozygous for *TSC *express elevated cyclin D1 compared with unaffected kidney tissue [26]. In addition cortical tubers microdissected from TSC patients showed elevated cyclin D1 mRNA expression in giant cells [27]. Tumor cells showed overexpression of cyclin D1 but lacked the loss of heterozygosity of the *TSC1 *and *TSC2 *genes. The result suggests that the overexpression of cyclin D1 may play an important role in the tumorigenesis [28]. Mouse embryonic fibroblast cells with low copy of *TSC2 *gene express 4-fold higher cyclin D1 compared to wild type cells (data not shown). Cyclin D1 protein expression is 4-fold higher in angiomyolipomas kidney tissue of patients with TSC compared to control samples suggesting that partial loss of tuberin is sufficient to upregulate cyclin D1. In accordance with our observation, cortical tubers microdissected from TSC patients show elevated cyclin D1 mRNA expression in the malignant cells [29]. Accumulation of cyclin D1 together with cyclin-dependent kinases may enhance cell proliferation in tumor cells tissue and may promote cells transition from G0/G1 to S phase.

## Conclusion

Our data show that the increase in tuberin phosphorylation and the deficiency in tuberin expression are associated with decreased protein and mRNA expression of OGG1 in angiomyolipomas kidney tissue from TSC patients. Decrease OGG1 results in the accumulation of 8-oxodG in kidney tumors, suggesting that tuberin plays a significant role in protecting the cells from oxidative DNA damage. Mismatched DNA base lesions, a form of genomic instability that if left unrepaired promotes additional genetic alterations leading kidney tumor phenotype. Increased phosphoylation of S6Kinase at Thr389 in tumor kidney tissues indicated the increase in mTOR activity in tumor tissue. In addition, increase cyclin D1 expression in tumor samples suggested that partial loss of tuberin is sufficient to upregulate cyclin D1 that may enhance cell proliferation in tumor cells tissue.

## Materials and methods

### Kidney tissues

Kidney angiomyolipoma tissue from 20 TSC patients with renal angiomyolipoma and 18 unrelated healthy people were obtained from the Brain and Tissue Bank for Development Disorders (University of Maryland, Baltimore, Maryland, USA) and San Antonio Cancer Institute Core, San Antonio, TX. The study has been approved by the Institutional Review Board of The University of Texas Health Science Center at San Antonio, TX.

### Protein extraction and immunoblot analysis

Kidney homogenates were prepared using lysis buffer (1× PBS, 1% NP-40, 0.5% sodium deoxycholate, 0.1% SDS) containing the protease inhibitors phenylmethylsulfonyl fluoride (10 mg/ml), leupeptin (10 mg/ml), and aprotinin (20 mg/ml). Kidney homogenates were centrifuged at 14,000 *g *for 30 min at 4°C. Protein concentration was determined with the Bradford assay [30] using bovine serum albumin as a standard. Protein (100 μg) was subjected to SDS-polyacrylamide gel electrophoresis. Proteins were transferred to polyvinylidene difluoride (PVDF) membrane at a constant voltage of 200 V for 1–1.5 h. PVDF membranes were blocked in 5% nonfat dried milk in TBS-0.1% Tween buffer [25 mM Tris HCl, 0.2 mM NaCl, 0.1% Tween 20 (vol/vol), pH 7.6] (TBS-T) for 1 h. Membranes were incubated with the respective primary antibodies overnight at 4°C. Rabbit polyclonal antibody raised against human OGG1 protein was generously provided by Dr. S. Mitra (University of Texas Medical Branch at Galveston, Texas). Phosho-tuberin, tuberin, phospho S6K, S6K were purchased from Cell Signaling Technology (Danvers, MA). Rabbit anti-NF-YA and cyclin D1 antibodies were purchased from Santa Cruz Biotechnology. All primary antibodies were prepared at 1:1,000 dilutions in TBST. Membranes were washed 3× with TBST and then incubated with an appropriate horseradish peroxidase-conjugated secondary antibody for 1 h at room temperature. An enhanced chemiluminescence kit (Amersham) was used to identify protein expression. Membranes were stripped with 0.2 M NaOH for 10 min each, blocked with 5% milk for 1 h, and then incubated with the respective primary and secondary antibodies, as described earlier. Expression of each protein was quantified by densitometry using National Institutes of Health Image 1.62 software.

### mRNA analysis by RT-PCR analysis

RNA was extracted from kidney tissue of control and tumors samples using RNeasy Mini kit (Qiagen, Valencia, CA). RNA was quantitated by spectrophotometery at 260 nm, and its integrity tested by formaldehyde/agarose gel electrophoresis. First strand synthesis of cDNA was carried in 20 μl total reaction volume as follows: 1 μl oligo [23]_12–18_, 2 μg total RNA, 1 μl of 10 mM dNTP mix (10 mM each dATP, dGTP, dCTP and dTTP) and 12 μl distilled water. The mixture was heated at 65°C for 5 min and then quickly chilled on ice. The contents of the tube were collected by brief centrifugation then 4 μl 5× first-Strand buffer, 2 μl 0.1 M DTT and 1 μl RNaseOUT Recombinant Ribonuclease Inhibitor (40 units/μl). The mixture was incubated at 42°C for 2 min and then 1 μl of SUPERSCRIPT II (200 U) was added and mixed by pipetting. The reaction mixture was incubated for 50 min at 42°C and then was inactivated by heating at 94°C for 2 min. PCR reaction was carried out in a final volume of 50 μl as follow: 5 μl 10 × PCR buffer, 1.5 μl 50 mM MgCl_2_, 1 μl 10 mM dNTP, 0.4 μl Taq DNA polymerase (5 U/μl), 2 μl cDNA (from first-strand reaction) 1 μl each of OGG1 upstream/reverse primers (5'AACATTGCTCGCATCACTGGC/5'GATGTCCACAGGCACAGCCTG) or GADPH upstream/reverse primers (5'ACCACAGTCCATGCCATCA/5'TCCACCACCCTGTTGCTGTA) and 38.1 μl autoclaved distilled water. The amplification for OGG1 and GADPH was carried out using a thermocycler (Biometra) programmed for 25 cycles consisting of 95°C (5 min), 95°C (1 min), 52°C (50 sec), 72°C (1 min) and 72°C (10 min) and yielded 356 and 495 bp, respectively of amplified products. The amplified PCR products were separated on 2% agarose gels. PCR products were analyzed on ethidium bromide stained gel. The yield was integrated for each sample on image analyzer and the ratio of OGG1 to GAPDH then calculated.

### 8-OxodG assay

DNA was isolated from the kidney tissue and detection of dG and 8-oxodG was performed on DNA hydrolyzed with nuclease P1 and alkaline phosphatase as previously described and validated [21]. Aliquots (90 μl) of DNA hydrolysates were injected onto a Partisil 5- m ODS-3 reverse-phase analytical column for HPLC analysis with the eluate monitored with a UV photodiode array (Shimadzo, SPD M10A) and electrochemical (EC) detectors (ESA Coul Array). Authentic standards of 8-oxodG and dG were analyzed along with every batch of samples. Salmon sperm DNA (5–50 μg) was used as a positive control for DNA digestion reactions. Standard curves for dG and 8-oxodG were prepared and quantitation was performed by linear regression analyses. Data were expressed as picomoles of 8-oxodG/dG × 10^-5 ^in 90 μl of DNA hydrolysate.

### Immunostaining of phospho-S6k

Phospho-S6K expression was also assessed by immunofluorescence histochemistry as previously described [22]. Acetone-fixed frozen kidney sections (4 μm) were incubated with nonimmune donkey IgG to block nonspecific binding, then incubated with rabbit anti- Phospho-S6K antibody followed by fluorescene isothiocyanates (FITC) FITC-labeled donkey anti-rabbit IgG (Chemicon International, Inc., Temecula, CA, USA) as secondary antibodies for signal detection. All incubations of primary and secondary antibodies were for 30 minutes with three washes with phosphate-buffered saline (PBS) containing 0.1% bovine serum albumin (BSA), 5 minutes each between steps. Controls consisted of PBS/BSA in place of primary antibody followed by detection procedures as outlined above. Kidney sections were viewed and photographed using an Olympus Research microscope equipped for epifluorescence with excitation and band pass filters. FITC green signals for phospho-S6k was detected using a filter with excitation at 535 nm.

### Statistics

Data are presented as mean ± standard error. Statistical differences were determined using ANOVA followed by Student Dunnett's (Exp. vs. Control) test using 1 trial analysis. *P-*values less than 0.05 and 0.01 were considered statistically significant.

## Abbreviations

TSC2: tuberous sclerosis complex-2; 8-oxodG: 8-oxodeoxyguanine; OGG1: 8-oxoG-DNA glycosylase; RCC: renal cell carcinoma.

## Competing interests

The author declares that they have no competing interests.

## Authors' contributions

SLH conceived the concept, designed the study, and prepared the manuscript. SLH obtained the samples and performed the western blotting, immunohistochemistry and extracted the DNA for 8-oxodG measurements.
